# Effect of Short-Term Grape Powder Supplementation in Patients with Crohn’s Disease: A Pilot Study

**DOI:** 10.3390/nu18121844

**Published:** 2026-06-08

**Authors:** Mohammad Shahir Eftekhar, Drishtant Singh, Jeffry Katz, Vu Nguyen, Paola Menghini, Alexander Rodriguez-Palacios, Fabio Cominelli, Abigail Raffner Basson

**Affiliations:** 1 Department of Surgery, School of Medicine, Shahid Beheshti Hospital, Qom University of Medical Sciences, Qom 37169-87366, Iran; shahiranthony@gmail.com; 2 Division of Gastroenterology & Liver Diseases, Case Western Reserve University School of Medicine, 2109 Adelbert Road, Biomedical Research Building 9th Floor, Cleveland, OH 44106, USA; dxs1087@case.edu (D.S.); jeffry.katz@uhhospitals.org (J.K.); vu.nguyen@uhhospitals.org (V.N.); pxm295@case.edu (P.M.); axr503@case.edu (A.R.-P.); fabio.cominelli@uhhospitals.org (F.C.); 3 Digestive Health Research Institute, Case Western Reserve University School of Medicine, 2109 Adelbert Road, Biomedical Research Building 9th Floor, Cleveland, OH 44106, USA; 4 Department of Nutrition, Case Western Reserve University School of Medicine, 2109 Adelbert Road, Biomedical Research Building 9th Floor, Cleveland, OH 44106, USA; 5 Digestive Health Institute, University Hospitals Cleveland Medical Center, 11100 Euclid Avenue, Cleveland, OH 44106, USA

**Keywords:** Crohn’s disease, inflammatory bowel disease, freeze-dried grape powder, gut microbiota, fecal myeloperoxidase, microbiome

## Abstract

**Background**: The overall objective of this pilot diet intervention study was to determine the effect of grape powder (GP) supplementation on gut microbiota composition and inflammatory markers in individuals with Crohn’s disease (CD). **Methods**: Adult CD participants were recruited from the Digestive Health Institute at University Hospitals Medical Center, Cleveland. All participants were supplemented with 45 g/day of freeze-dried grape powder (equivalent to ~1.5 cups of fresh grapes) daily for 21 days. The primary outcome was the change in fecal microbiome profiles. Secondary outcomes included the absolute difference (day 21-day 0) in Harvey Bradshaw Index (HBI) score, fecal myeloperoxidase (MPO), and high-sensitivity C-reactive protein (hsCRP). **Results**: A total of 21 CD participants were included in the final analysis. After 21 days of GP supplementation, more than half of the participants (13, 61.9%) experienced a reduction in fecal MPO, while 80% (17) experienced either a reduction or no change in HBI score. Microbiome analysis revealed modest but directional shifts, including enrichment of *Akkermansiaceae*, *Bacteroidaceae*, *Tannerellaceae*, *Rikenellaceae*, and *Monoglobaceae*. While overall community structure did not significantly change at the cohort level, individualized microbiome responses as well as functional pathway shifts were observed following the intervention. **Conclusions**: Daily supplementation with freeze-dried grape powder for 21 days was safe and well-tolerated in adults with CD and was associated with modest shifts in gut microbiome composition. This study was registered on clinicaltrials.gov (NCT05972694; 5 February 2024).

## 1. Introduction

The inflammatory bowel disease (IBD) subtype Crohn’s disease (CD) is a chronic, lifelong disorder of the gastrointestinal tract that is characterized by increased intestinal permeability and alterations in fatty acid and SCFA and oxidative stress [1,2,3]. While several factors, including genetics and environmental influences, are believed to contribute to the etiology of the disease, evidence indicates that the gut microbiome plays a key role in both disease onset and progression [4]. Dietary factors play a crucial role in shaping the composition of the gut microbiota and, as such, are being extensively explored for their potential to therapeutically modulate the microbiome to help attenuate both the symptoms and progression of CD [5,6,7].

Grapes contain a wide variety of phytochemicals and polyphenols with strong antioxidant properties (e.g., proanthocyanidins, anthocyanins, leucoanthocyanidin, quercetin, kaempferol, stilbenes, ellagic acid, hydroxycinnamates), as well as provide a rich source of dietary fiber [8]. Recent evidence shows that grape-derived products (e.g., polyphenols) improve endothelial function [9], reduce blood cholesterol and low-density lipoprotein (LDL) oxidation in cardiovascular disease [10,11,12], decrease inflammatory biomarkers [13], and reduce oxidative stress [14] in humans. These effects may be partly mediated by alterations in the gut microbiota [15,16]. Both animal and human studies demonstrate that grapes and grape-derived polyphenols can modify microbial composition, while the microbiota, in turn, metabolize these compounds, increasing the production of bioactive polyphenolic metabolites [16]. Specifically, grape-derived compounds are metabolized by gut bacteria into various phenolic metabolites, beneficial polyunsaturated fatty acids such as docosahexaenoic acid (DHA) and short-chain fatty acids (SCFA), which have shown beneficial effects in humans, for instance, those affected with CVD and cancer [15,16]. Recently, laboratory animals relevant to IBD have shown that these key metabolites could improve gut barrier function and intestinal immunity, and exert anti-inflammatory activity in human IBD patients [15,16,17,18,19]. Because increased intestinal permeability and alterations in fatty acid and SCFA and oxidative stress are associated with IBD [1,2,3], we hypothesized that grape consumption could induce beneficial changes in the gut microbiota, as well as reduce intestinal inflammation and improve symptoms in patients with CD.

To evaluate the effects of grapes on the gut microbiota and inflammatory profiles in CD, we conducted a pilot dietary intervention to assess whether daily consumption of freeze-dried grape powder (GP) for 21 days alters microbiota composition and improves disease symptoms and inflammatory markers in adults with CD.

## 2. Materials and Methods

### 2.1. Study Setting and Participants

This open-label study evaluated the effect of freeze-dried GP consumed daily for 21 days in adult patients with CD. Between September 2024 and August 2025, CD outpatients were recruited during routine visits at the Digestive Health Institute (DHI) at University Hospitals Cleveland Medical Center (UHCMC).

### 2.2. Inclusion and Exclusion Criteria

Male and female adult patients (aged 18–65 years) with CD established by standard endoscopic, histologic, and radiologic criteria, with a Harvey Bradshaw Index (HBI) score of <6, able to take oral nutrition, and with internet access to complete study activities were included. Exclusion criteria were pregnancy/lactation, short bowel syndrome, hospitalized patients, body mass index < 16 kg/m^2^ or >35 kg/m^2^, individuals who lack consent capacity, known drug abuse, known parasitic disease of digestive system, ostomy, known symptomatic intestinal stricture, known berry or grape allergy, change in IBD-related medication or documented *Clostridium difficile* infection within 4 weeks of screening, probiotic or antibiotic use for the previous 14 days, history of <1 bowel movements per day.

### 2.3. Grape Powder Intervention

Participants were supplemented with 45 g/d freeze-dried grape powder (equivalent to approximately 1.5 cups of fresh grapes) consumed as 22.5 g packets twice daily. Each sachet was dissolved in 8 oz of water, with one taken in the morning and one in the evening with meals. The GP used for this study was obtained from the California Table Grape Commission (Fresno, CA, USA). The phytochemical, nutrient, compositional and microbiological analysis of the GP is provided in Supplementary Tables S1–S4. Participants were instructed to avoid all berries and berry-containing foods or beverages beginning seven days prior to initiation of the GP intervention and continuing throughout the intervention period. During the study, participants were asked to maintain their usual diet and physical activity patterns and to avoid initiating new supplements or making substantial dietary changes.

### 2.4. Recruitment

Participants were identified using appointment lists from the DHI at UHCMC and through the University Hospitals electronic medical record system (TriNetX). Recruitment emails and hospital advertisement flyers were also used. Potentially eligible individuals were pre-screened during their routinely scheduled appointment with their treating gastroenterologist.

### 2.5. Outcome Measures

Primary and key secondary outcomes were measured on day 0 (d0) of the GP intervention and again on day 21 (d21) of the GP intervention. The primary outcome was the change in 16S fecal microbiome profiles. Secondary outcomes included the absolute difference (d21–d0) in body weight, HBI score, fecal myeloperoxidase (MPO) activity, and high-sensitivity C-reactive protein (hsCRP; <1.0 mg/L normal, >5 mg/L inflammation). The HBI is a clinical measure of disease activity and includes general well-being, abdominal pain, number of liquid stools per day, presence of an abdominal mass, and complications, with scores of ≤4 indicating remission, and a score > 8 indicating moderate disease. Fecal MPO was quantified at day 0 and day 21 of the intervention. Additional continuous outcome measures included the short Crohn’s disease activity index (sCDAI), assessed daily via REDCap survey, starting 7 days prior to starting the study diet, through the study duration. The sCDAI is a validated tool that assesses patient-reported outcomes focusing on cardinal CD symptoms: diarrhea, abdominal pain, and general well-being.

### 2.6. Laboratory Analysis

Blood serum was collected via venipuncture in a serum separator tube and analyzed for hsCRP by UHCMC Laboratory Services. Freshly voided stool samples were stored at −80 °C within 2 h of collection. In brief, participants collected freshly voided fecal samples at home, using Covidien commode specimen collectors, which were then placed into sealable bags and a cooler containing three ice packs. A medical courier service was available to all participants for the transport of fecal samples to the laboratory for analysis. Samples were processed into 4 individual aliquots prior to storage at −80 °C. Fecal MPO activity was assessed (measured in triplicate) within 3 days of collection, as previously described [20,21].

### 2.7. Adherence and Safety Monitoring

All participants received standardized counseling from a registered dietitian on avoiding grape- and berry-containing foods to prevent inadvertent consumption during the study period. Daily grape powder intake was self-recorded by participants. Adherence was assessed by the research team through counts of returned sachets at the end of the study visit (day 21 of intervention). In addition, a self-reported 3-day food record was performed by participants halfway through the study to determine that no berry-containing foods were consumed. Adverse events were assessed at the end of the study visit. Serious adverse events included any adverse event that was fatal, life-threatening, requiring prolonged hospital stay, congenital anomaly or birth defect, or other medically significant event as deemed such by the investigator.

### 2.8. Ethical Considerations

This study was reviewed and approved by the institutional review board of UHCMC (STUDY20190080; initial approval 6 June 2023) and registered on clinicaltrials.gov (NCT05972694; 5 February 2024). All patients provided written informed consent before study entry. All authors had access to the study data and reviewed and approved the final manuscript.

### 2.9. Microbiome Analysis

DNA from fecal samples was isolated using the QIAGEN DNeasy PowerSoil Pro Kit (Qiagen, Valencia, CA, USA), according to the manufacturer’s protocol. Isolated DNA was quantified using Qubit Flex fluorometer (Thermofisher Scientific, Waltham, MA, USA) and Qubit™ dsDNA HS Assay Kit (Thermofisher Scientific, Carlsbad, CA, USA). DNA libraries were prepared using the Quick-16S Plus NGS Library Preps kit (V3V4) (Zymo Research, Irvine, CA, USA). Target regions were amplified via PCR using the following primers: 341f (CCTACGGGDGGCWGCAG, CCTAYGGGGYGCWGCAG, 17 bp) and 806R (GACTACNVGGGTMTCTAATCC, 24 bp), which cover hypervariable regions V3 and V4. The forward primer 341f is a mixture of the two sequences listed. The 10-nt dual-indices, which are included with the kit, were subsequently added by PCR. Final libraries were quantified using Qubit Flex fluorometer and quality was assessed by TapeStation D1000 ScreenTape (Agilent Technologies Inc., Palo Alto, CA, USA). DNA libraries were quantified using a Qubit Flex fluorometer and Qubit™ dsDNA HS Assay Kit. Libraries were then circularized using the Element Adept library compatibility workflow and sequenced on the Element AVITI platform using the AVITI 2x300 Cloudbreak sequencing kit (Element Biosciences, San Diego, CA, USA).

The unassembled sequencing reads were processed and analyzed through the CosmosID bioinformatics platform (CosmosID Inc., Germentown, MD, USA) The CosmosID-HUB Microbiome’s 16S workflow implements the DADA2 algorithm [22] as its core engine and utilizes the Nextflow ampliseq pipeline [23] definitions to run it on our cloud infrastructure. Briefly, primer removal is done with Cutadapt [24], and quality trimming parameters are passed to DADA2 to ensure that the median quality score over the length of the read exceeds a certain Phred score threshold. Within DADA2, forward and reverse reads are each trimmed to a uniform length based on the quality of reads in the sample; higher quality data will generally result in longer reads. DADA2 uses machine learning with a parametric error model to learn the error rates for the forward and reverse reads, based on the premise that correct sequences should be more common than any particular error-variant. DADA2 then applies its core sample inference algorithm to the filtered and trimmed data, applying these learned error models. Paired-end reads are then merged if they have at least 12 bases of overlap and are identical across the entire overlap. The resulting table of sequences and observed frequencies is filtered to remove chimeric sequences (those that exactly match a combination of more-prevalent “parent” sequences). Taxonomy and species-level identification (where possible) are conducted with DADA2’s naive Bayesian classifier, using the Silva version 138 database.

Lastly, the predicted functional potential of the community was profiled using PICRUST2 [25]. Briefly, PICRUSt2 (Phylogenetic Investigation of Communities by Reconstruction of Unobserved States) is a tool that predicts functional capabilities and abundances of a microbial community based on the observed amplicon (marker gene) content. Functional capabilities are given by EC classifiers or MetaCyc ontologies, and these can be aggregated to predict pathways that are likely present in a given sample.

### 2.10. Statistical Analysis

Descriptive data are reported as mean and SD or as counts and percentages. Formal statistical comparisons were performed comparing the baseline (d0) and post-study (d21) intervention data using two-tailed paired *t*-tests and the Wilcoxon rank sum test based on data normality. We included both male and female participants. Although blinding of study participants and evaluators to treatment assignment was not feasible, all sample analyses and statistical evaluations were conducted in a blinded fashion. A *p*-value < 0.05 was considered statistically significant. Analyses were performed using GraphPad Prism v10.0 (GraphPad Software, San Diego, CA, USA).

## 3. Results

### 3.1. Participants

In total, 700 individuals with CD were contacted via a recruitment letter. Of these, 113 (16%) expressed interest, of whom 31 (27.4%) were ineligible, and 74 (65.4%) either chose not to participate or were unable to be contacted. Of the 29 CD patients who signed informed consent, 7 (24.1%) were lost to follow-up prior to attending study visit 1, and 1 withdrew due to an adverse event. A total of 21 CD individuals completed the study and were included in the final analysis.

Baseline demographic, disease, and clinical characteristics of CD participants are shown in Table 1. Overall, 52.3% (11) of the participants were women, and the majority of participants were White, followed by African American. All participants had a college education or higher, and the median body mass index at screening was 26.78 kg/m2 (IQR 24.55, 30.00 kg/m^2^). The mean number of years since diagnosis was 18.7 ± 12.0, with 39.0% (8) having non-stricturing/non-penetrating disease compared to 19.0% (4) having penetrating disease and 9.5% (2) having a history of structuring disease. At enrollment, participants had a mean HBI score of 1.71 ± 1.92, with the majority (16/21, 76%) taking immunosuppressive/biologic medications at the time of enrollment.

Paired analysis showed no significant difference in body weight from day 0 to day 21 (d0: 171.8 ± 36.23 vs. d21: 170.4 ± 39.35, *p* = 0.23).

### 3.2. Effect on Symptoms and Markers of Inflammation

**Figure 1:**
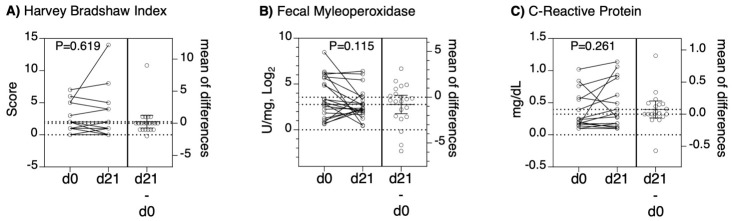
Effect of grape powder on disease symptoms and inflammatory markers in patients with Crohn’s disease. The right panel shows paired analysis of the change in disease symptoms and inflammation from day 0 to day 21. Individual participant values are shown with paired lines connecting d0 and d21. The right panels display the mean difference (d21–d0) with corresponding distributions. (**A**) Harvey Bradshaw Index; HBI, (**B**) fecal myeloperoxidase; MPO (log_2_-transformed), and (**C**) serum C-reactive protein; CRP. CRP excludes two participants due to insufficient blood drawn on one occasion.

The change in fecal MPO, hsCRP, and HBI score of CD participants at the start of the GP intervention (day 0) and day 21 of the intervention is shown in Figure 1. Overall, a total of 7 (33.3%) participants experienced a reduction in HBI score, 4 (19.0%) experienced an increase, and 10 (47.6%) experienced no change in HBI score (d0: 1.81 ± 2.15 vs. 2.04 ± 3.47, Paired *t*-test *p* = 0.619, Figure 1A). More than half of the participants (13, 61.9%) experienced a reduction or no change (3, 14.2%) in fecal MPO compared to 5 (23.8%) having an increase (d0: 3.55 ± 2.47 vs. d21: 2.76 ± 1.59, Paired *t*-test *p* = 0.115, Figure 1B). Although hsCRP concentrations on day 0 and day 21 were within a threshold of 5 mg/dL, overall, 10 (47.6%) participants experienced a reduction or no change in hsCRP value, compared with 9 (42.8%) who experienced an increase (d0: 0.34 ± 0.28 vs. d21: 2.04 ± 3.47, Paired *t*-test *p* = 0.261, Figure 1C).

### 3.3. Adherence to the GP Intervention

Overall, only one participant reported missing a single day of GP consumption, and no participants reported consuming any additional grape-containing foods or beverages during the intervention period.

### 3.4. Adverse Events

Daily consumption of the GP was well-tolerated over the 21 days. One participant reported abdominal pain and nausea following GP consumption; these symptoms resolved within three hours of ingestion. No serious adverse events were reported.

### 3.5. Microbiome Composition

**Figure 2:**
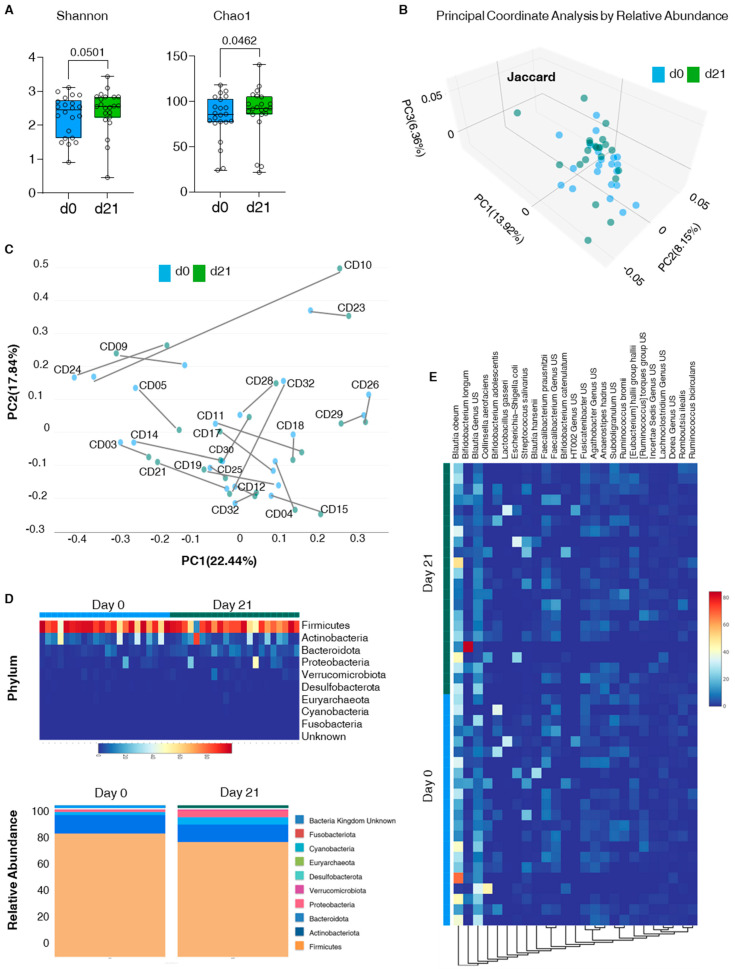
Effects of grape powder supplementation on gut microbiome diversity and composition in response to Grape Powder. (**A**) Alpha diversity indices, (**B**) principal coordinate analysis (PCoA) based on relative abundance data, (**C**) Bray–Curtis dissimilarity metrics, and (**D**) top panel: heatmap showing the relative abundance of bacterial phyla across individual samples collected at Day 0 and Day 21. Bottom panel: Stacked bar plots summarizing the mean relative abundance of bacterial phyla at each time point. (**E**) Heatmap shows a change in the top 25 most abundant species.

We conducted 16S rRNA Amplicon sequencing of pre- and post-fecal samples to determine how the grape powder affects the gut microbiome profiles. Relative abundance alpha diversity indices (Shannon/Simpson diversity) showed significant differences in the pre (d0) and post (d21) fecal samples due to the grape powder (Figure 2A). While principal coordinate analysis (PCoA; Jaccard and Bray–Curtis dissimilarity) showed no substantial change in overall microbiome structure after 21 days of GP exposure (Figure 2B,C), noticeable shifts were seen at the individual level (Figure 2C), suggesting that GP elicits individualized changes in microbial community composition rather than uniform effects across participants.

**Figure 3:**
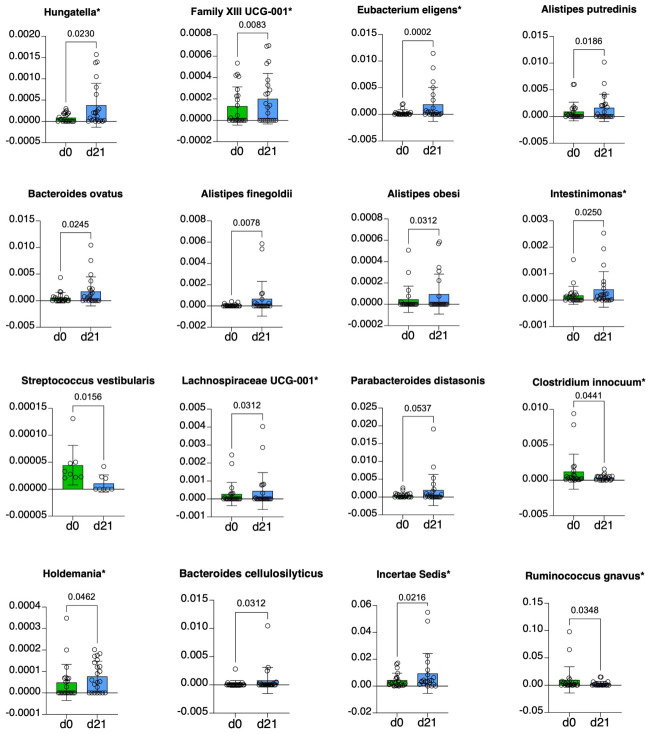
Significant species-level caxa Comparing day 0 to day 21. Species-level analysis revealed 16 species that were significantly different from day 0 to day 21 across the CD participants. *p*-value represents the Wilcoxon matched-pairs signed rank test. * denotes ‘group Genus Unknown Species’.

Overall, at the phylum level, gut microbial composition of the CD participants prior to the GP intervention was enriched in *Firmicutes* and *Actinobacteria*, followed by *Bacteroidota*, *Proteobacteria*, and *Verrucomicrobiota* (Figure 2D). After 21 days of GP supplementation, there was a significant enrichment in *Bacteroidota* and *Verrucomicrobiota* across the CD participants. At the family level, this was represented by significant enrichments in *Akkermansiaceae*, *Bacteroidaceae*, *Tannerellaceae*, *Rikenellaceae*, and *Monoglobaceae* (Bacillota, formerly Firmicutes, Supplementary Figure S1). Species-level analysis revealed 16 species that were significantly different from day 0 to day 21 across the CD participants (representing approximately 16/419; 3.81% of the total number of species analyzed per group, Figure 2E and Figure 3).

**Figure 4:**
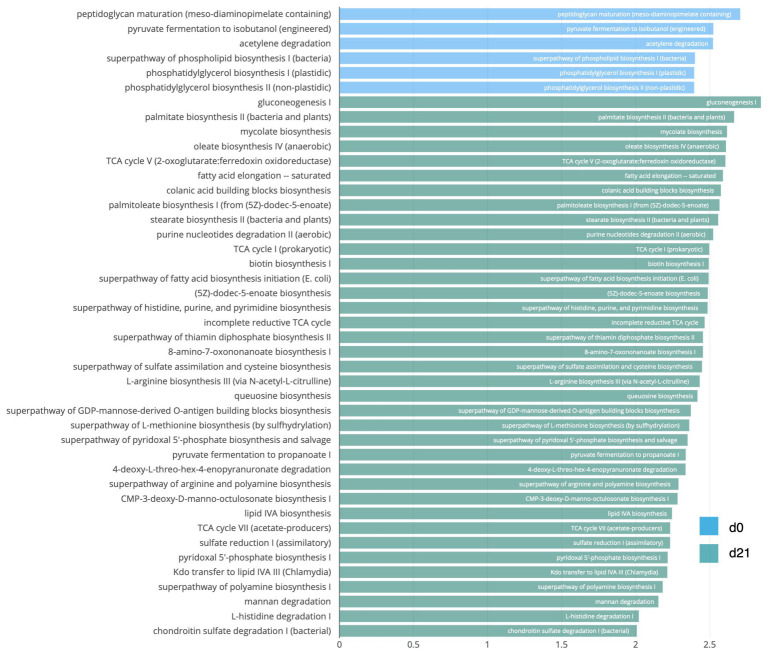
Differentially abundant microbial functional pathways identified by LEfSe analysis comparing d0 and day 21. Bars represent linear discriminant analysis (LDA) scores for pathways that significantly differed between timepoints. Pathways enriched at d0 are shown in blue, and those enriched at d21 are shown in green. Only significantly discriminative features are included based on LEfSe criteria.

Linear discriminant analysis of effect size (LeFse) was used to identify functional pathways that differed significantly between CD participants, comparing day 0 to day 21. Pathways differentially enriched are shown in Figure 4.

## 4. Discussion

This study is the first to evaluate the effects of daily GP consumption for 21 days on the gut microbiome and clinical markers of inflammation in patients with CD. Overall, GP supplementation was well tolerated by CD participants. While the intervention did not significantly reduce fecal or systemic markers of inflammation, over half of the participants demonstrated a reduction in fecal MPO from day 0 to day 21. This is clinically relevant, as patients who are considered to be in remission remain at elevated risk of flare due to persistent subclinical inflammation [26,27], and even modest reductions in neutrophil-associated markers such as MPO may meaningfully influence disease trajectories.

Microbiome analysis showed that, although the GP intervention produced modest overall shifts at the cohort level, noticeable within-individual changes were observed, suggesting that GP-induced microbiome shifts depend on baseline microbial composition. Overall, at the family level, 21 days of GP supplementation resulted in increased abundance of several taxa typically associated with anti-inflammatory effects and often reduced in CD, namely *Akkermansia*, *Tannerellaceae*, and *Rikenellaceae*. Of interest, GP also increased abundance in certain rare taxa, namely *Monoglobaceae*, a fiber-degrading (fibrolytic) taxon that has recently emerged as an important contributor to the gut ecosystem health with potential protective roles against inflammation [28,29]. At the species level, GP induced shifts in taxa associated with a healthier, fiber-fermenting gut ecosystem, as well as taxa reported to exhibit context-dependent roles in CD. For example, GP increased the abundance of *Eubacterium eligens*, a butyrate-producing species [30,31] often depleted in CD patients, contributing to gut dysbiosis and inflammation. In comparison, GP also increased the relative abundance of taxa with context-dependent effects, including *Hungatella* Genus unknown species [32,33], *Alistipes Putendris* [34,35], *Bacteroides ovatus* [36,37,38], and *Family XIII UCG-001* [39]. Although some strains have been associated with pro-inflammatory effects, the overall body of evidence supports beneficial, anti-inflammatory roles, including contributions to intestinal barrier integrity. Collectively, these findings support a GP-induced shift toward a less inflammatory microbial community, although the literature is mixed for some taxa and often genus- rather than species-level.

Functional pathway analysis (LEfSe) revealed distinct shifts in microbial metabolic activity after 21 days of GP supplementation. At baseline (day 0), enrichment of pathways related to bacterial structural components and membrane biosynthesis, which have been previously linked to microbial virulence, epithelial stress, and inflammation in IBD, was identified. These included peptidoglycan maturation [40,41,42], phospholipid biosynthesis [43], lipopolysaccharide (LPS)-associated pathways (e.g., CMP-KDO biosynthesis) [44,45,46], and fatty acid biosynthesis and nucleotide degradation [40]. In comparison, post-intervention samples (day 21) showed enrichment in pathways involved in amino acid, polyamine, and vitamin biosynthesis, including arginine/polyamine [47,48,49] metabolism [50,51] and biotin biosynthesis [52,53,54], which have been associated with epithelial repair, immune regulation, and maintenance of mucosal homeostasis. These findings are consistent with a shift toward a more metabolically favorable microbial functional profile.

This pilot study has several limitations. First, the short intervention duration and absence of a control group limit the generalizability of the findings and preclude definitive attribution of observed effects to the GP intervention. Second, dietary intake outside of the intervention was not controlled, which may have contributed to inter-individual variability in microbiome responses. Third, the open-label design may introduce bias in patient-reported outcomes, although the use of objective inflammatory markers partially mitigates this limitation. Finally, microbiome analyses were based on 16S rRNA sequencing, which limits taxonomic resolution and provides inferred functional insights.

## 5. Conclusions

Daily supplementation with freeze-dried grape powder for 21 days was safe and well tolerated in adults with CD and was associated with modest, individualized shifts in gut microbiome composition, without worsening clinical disease activity or inflammatory markers. Larger, controlled studies over a longer period may be warranted to determine the clinical relevance and mechanistic implications of these findings.

## Figures and Tables

**Table 1:** Baseline characteristics of Crohn’s disease participants.

Characteristic	Crohn’s, *N* = 21
Age at randomization, y	45.2 ± 14.8
Female sex, *n* (%)	11 (52.3)
Race, *n* (%)	
White	19 (90.5)
Black	2 (9.5)
Marital status, *n* (%)	
Married	13 (62)
Single/Divorced	8 (38)
Education, *n* (%)	
College	10 (47.6)
Graduate	11 (52.4)
BMI, kg/m^2^	27.22 ± 4.91
Years since Crohn’s diagnosis	18.7 ± 12.0
CD behavior, *n* (%)	
Non-stricturing, non-penetrating	8 (38.0)
Stricturing	4 (19.0)
Penetrating	9 (42.8)
Stricturing and penetrating	2 (9.5)
CD distribution, *n* (%)	
Ileum alone	11 (52.3)
Colon alone	4 (19.0)
Ileum and colon	6 (28.5)
History of perianal disease, *n* (%)	2 (9.5)
History of intestinal surgery, *n* (%)	6 (28.5)
Current IBD medications, *n* (%)	
adalimumab	4 (19.0)
azathioprine	1 (4.7)
infliximab	2 (9.5)
risankizumab	2 (9.5)
sulfasalazine	2 (9.5)
ustekinumab	1 (4.7)
upadacitinib	2 (9.5)
vedolizumab	1 (4.7)
6-mercaptopurine	1 (4.7)
5-aminosalicylic acid (5-ASA)	1 (4.7)
oral corticosteroids	1 (4.7)
hsCRP ^1^, mg/dL	0.49 ± 0.74
HBI ^2^ score	1.71 ± 1.92

^1^ hsCRP; high sensitivity C-reactive protein, ^2^ HBI; Harvey Bradshaw Index. The values represented as Mean ± SD or *n* (%).

## Data Availability

The original contributions presented in the study are included in the article/Supplementary Materials; further inquiries can be directed to the corresponding author. De-identified individual participant data are available indefinitely at figshare 10.6084/m9.figshare.31964730.

## Supplementary Materials

The following supporting information can be downloaded at: https://www.mdpi.com/article/10.3390/nu18121844/s1, Table S1. Phytochemicals Analyzed in Freeze-Dried Preparation (Y2018). Table S2. Nutrient Analysis of Freeze-Dried Table Grape Powder (Y2018). Table S3. Compositional Analysis of Freeze-Dried Table Grape Powder (per 24 g). Table S4 Microbiological Analysis of Freeze-Dried Table Grape Powder (Y2018). Figure S1. Family-level analysis of taxa significantly different from day 0 to day 21 across the CD participants. *p*-value represents Wilcoxon matched-pairs signed rank test.

## Abbreviations

The following abbreviations are used in this manuscript:

CD	Crohn’s disease
DHI	Digestive Health Institute
GP	Grape powder
HBI	Harvey Bradshaw Index
hsCRP	high-sensitivity C-reactive protein
IBD	Inflammatory bowel disease
MPO	Myeloperoxidase
sCDAI	short Crohn’s disease activity index
SoyP	Soy-pea protein
UC	Ulcerative colitis
UHCMC	University Hospitals Cleveland Medical Center
