# Supplementary material for: Effect of Short-Term Grape Powder Supplementation in Patients with Crohn’s Disease: A Pilot Study
Source: Nutrients. 2026 Jun 8;18(12):1844. doi: 10.3390/nu18121844 (PMC13304518; doi:10.3390/nu18121844)
Supplement: Supplementary file 1 [file nutrients-18-01844-s001.zip › nutrients-4301038-supplementary.pdf]

SUPPLEMENTARY MATERIALS

Effect of Short-Term Grape Powder Supplementation in Patients with Crohn's Disease: A Pilot Study

Mohammad Shahir Eftekhar[1], Drishtant Singh[2,3], , Jeffry Katz[2,3,4], Vu Nguyen[2,3,4], Paola Menghini[2,3], Alexander Rodriguez-Palacios[2,3], Fabio Cominelli[2,3,4], Abigail Raffner Basson[1,2,3,4*]

[1] Department of Surgery, School of Medicine, Shahid Beheshti Hospital, Qom University of Medical Sciences, Qom, Iran.
[2] Division of Gastroenterology & Liver Diseases, Case Western Reserve University School of Medicine, Cleveland, OH, USA.
[3] Digestive Health Research Institute, University Hospitals Cleveland Medical Center, Cleveland, OH, USA.
[4] Digestive Health Institute, University Hospitals Cleveland Medical Center, Cleveland, OH, USA.
[5] Department of Nutrition, Case Western Reserve University School of Medicine, Cleveland, OH, USA.

Correspondence:
Abigail Raffner Basson (axb860@case.edu or 0829ab@gmail.com)
2109 Adelbert Road, Biomedical Research Building 9th floor, Cleveland, OH, 44106
Tel: +1(216) 630-0365

TABLE OF CONTENTS

A. **Supplementary Materials**

B. **Supplementary Tables**
Table S1. Phytochemicals Analyzed in Freeze-Dried Preparation
Table S2. Nutrient Analysis of Freeze-Dried Table Grape Powder
Table S3. Compositional Analysis of Freeze-Dried Table Grape Powder (per 24g)
Table S4. Microbiological Analysis of Freeze-Dried Table Grape Powder

C. **Supplementary Figure**
Figure S1. Family-level analysis of taxa significantly different from day 0 to day 21 across the CD participants.

A. Supplementary Materials

Fresh grapes

Fresh grapes contain about 82% water, 12-18% sugar, and 0.2-0.8% acid, mainly tartaric and malic acid. Grapes also contain numerous phenolic compounds, including simple phenols, simple phenolic acids, cinnamic acids, stilbenes, flavonoids, flavans, flavonols and anthocyanins. A standard serving size of fresh grapes is approximately 3/4 cup (126 grams).

Grapes are high in flavonoids and are particularly good sources of flavans. For example, the major phenolic compound in grapes is catechin, and they contain epicatechin, gallocatechin and epigallocatechin. Grapes also contain high concentrations of leucoanthocyanidin flavans of varied structure. Grapes are good sources of flavonols, primarily quercetin. Red and black grapes contain high amounts of anthocyanins.

Freeze-dried grape powder

The freeze-dried grape powder was produced from a composite of commercially grown fresh red, green, and black California grapes (Vitis vinifera; seeded and seedless varieties), representing those commonly available to consumers. Whole grapes were frozen, ground with food-grade dry ice, freeze-dried, and re-ground under Good Manufacturing Practices to preserve the integrity of biologically active compounds. The final product contains key grape phytochemicals, including resveratrol, flavans (e.g., catechin), flavonols (e.g., quercetin), anthocyanins, and simple phenolics. Silicon dioxide was added as an anti-caking agent, and the powder was stored moisture-proof containers at -70 °C until shipment to the investigator.

To estimate the amount of fresh grapes represented by the powdered preparation, moisture content must be taken into account. The dry powder contains approximately 1% moisture and fresh grapes contain approximately 82% moisture. Therefore, 100 grams of fresh grapes corresponds to approximately 18.2 grams powder.

B. Supplementary Tables

Supplementary Table S1. Phytochemicals Analyzed in Freeze-Dried Preparation

Compounds		Amount (per 100 g powder)
**Catechins**		
	Catechin	14.6 mg/kg +/- 0.718
	Epicatechin	2.43mg/kg +/- .12
**Anthocyanins**		
	Peonidin	2.62 mg/kg +/- .152
	Cyanidin	.24 mg/kg + /- .013
	Malvidin	6.96 +/- .298
**Flavonols**		
	Kaempferol	.139 mg/kg +/- .018
	Isorhamnetin	.145 mg/kg +/- .016
**Stilbenes**		
	Resveratrol	.33 mg/kg +/- .061
**Total Polyphenols** (in gallic acid equivalents)		354 mg/100g

This analysis does not represent the complete phytochemical profile of grapes.

Supplementary Table S2. Nutrient Analysis of Freeze-Dried Table Grape Powder

Nutrient	Amount (per 100 g powder)	Units
Calories	360	kcals
Total Fat, acid hydrolysis	.87	g
Total Carbohydrate (sugars)	82	g
Protein (N x 6.25)	3.7	g
Calcium	58.7	mg
Iron	1.78	mg
Sodium	32.4	mg
Potassium	1030	mg
Thiamin	.18	mg
Folic Acid	15.5	mcg
Phosphorus	100	mg
Magnesium	36.8	mg
Zinc	.241	mg
Copper	.413	mg
Manganese	.457	mg
Moisture	5.89	g
Ash	3.3	g

Nutrient analyses were performed using standard methods of analysis.

Supplementary Table S3. Compositional Analysis of Freeze-Dried Table Grape Powder (per 24g)

Analysis	Result
**Caloric Calculations**	
Calories	87.2 Cal/Serving size
Calories from Fat	0.477 Cal/Serving size
Total Carbohydrate	21.7 g/Serving Size
**Fat by Acid Hydrolysis**	
Fat	0.1 g/Serving Size
**Fatty Acids Calculated as Triglycerides**	
Saturated Fatty Acids (Acid Form)	0.018 g/Serving Size
Total Cis Unsaturated Fatty Acids (Acid Form)	0.0329 g/Serving Size
Monounsaturated Fatty Acids (Acid Form)	0.0042 g/Serving Size
Polyunsaturated Fatty Acids (Acid Form)	0.0285 g/Serving Size
Trans Fatty Acids (Acid Form)	<0.002 g/Serving Size
Omega 3 Fatty Acids	0.0070 g/Serving Size
Omega 6 Fatty Acids	0.023 g/Serving Size
Omega 9 Fatty Acids	0.0041 g/Serving Size
Total Fatty Acids	0.0530 g/Serving Size
4:0 Butyric	<0.002 g/Serving Size
6:0 Caproic	<0.002 g/Serving Size
8:0 Caprylic	<0.002 g/Serving Size
10:0 Capric	<0.002 g/Serving Size
12:0 Lauric	<0.002 g/Serving Size
14:0 Myristic	<0.002 g/Serving Size
14:1 Myristoleic	<0.002 g/Serving Size
15:0 Pentadecanoic	<0.002 g/Serving Size
15:1 Pentadecenoic	<0.002 g/Serving Size
16:0 Palmitic	0.016 g/Serving Size
16:1 Palmitoleic	<0.002 g/Serving Size
17:0 Heptadecanoic	<0.002 g/Serving Size
17:1 Heptadecenoic	<0.002 g/Serving Size
18:0 Stearic	0.0030 g/Serving Size
9c 18:1 Oleic	0.0040 g/Serving Size
18:2 Linoleic	0.023 g/Serving Size
18:3 Gamma Linolenic	<0.002 g/Serving Size
**Fatty Acids Calculated as Triglycerides**	
18:3 Alpha Linolenic	0.0069 g/Serving Size
18:4 Octadecatetraenoic	<0.002 g/Serving Size
20:0 Arachidic	<0.002 g/Serving Size
20:1 Eicosenoic	<0.002 g/Serving Size
20:2 Eicosadienoic	<0.002 g/Serving Size
20:3 Eicosatrienoic (n3)	<0.002 g/Serving Size
20:3 Homogamma Linolenic (n6)	<0.002 g/Serving Size
20:4 Arachidonic (n3)	<0.002 g/Serving Size
20:4 Arachidonic (n6)	<0.002 g/Serving Size
20:5 Eicosapentaenoic	<0.002 g/Serving Size
21:5 Heneicosapentaenoic	<0.002 g/Serving Size
22:0 Behenic	<0.002 g/Serving Size
22:1 Erucic	<0.002 g/Serving Size
22:2 Docosadienoic	<0.002 g/Serving Size
22:3 Docosatrienoic	<0.002 g/Serving Size
22:4 Docosatetraenoic	<0.002 g/Serving Size
22:5 Docosapentaenoic (n3)	<0.002 g/Serving Size
22:5 Docosapentaenoic (n6)	<0.002 g/Serving Size
22:6 Docosahexaenoic	<0.002 g/Serving Size
24:0 Lignoceric	<0.002 g/Serving Size
24:1 Nervonic	<0.002 g/Serving Size
Total 18:1 trans	<0.002 g/Serving Size
Total 18:1 cis	0.0044 g/Serving Size
Total 18:2 trans	<0.002 g/Serving Size
Total 18:3 trans	<0.002 g/Serving Size

Supplementary Table S3. (continued). Compositional Analysis of Freeze-Dried Table Grape Powder (per 24g)

Analysis	Result
Cholesterol	<0.24 mg/Serving Size
**Total Dietary Fiber**	
Total Dietary Fiber	1.10 g/Serving Size
**Sugar Profile By Ion Chromatography**	
Fructose	10.4 g/Serving Size
Galactose	<0.02 g/Serving Size
Glucose	9.19 g/Serving Size
Sucrose	<0.02 g/Serving Size
Lactose	<0.02 g/Serving Size
Isomaltulose	<0.02 g/Serving Size
Maltose	<0.02 g/Serving Size
Total Sugar	19.6 g/Serving Size
**Protein (N x 6.25) Dumas Method**	
Protein	0.539 g/Serving Size
**Elements by ICP Emission Spectrometry (ICP-OES)**	
Calcium	18.6 mg/Serving Size
Iron	0.430 mg/Serving Size
Sodium	4.83 mg/Serving Size
Potassium	238 mg/Serving Size
Phosphorus	23.8 mg/Serving Size
Magnesium	9.41 mg/Serving Size
Zinc	0.0516 mg/Serving Size
Copper	0.116 mg/Serving Size
Manganese	0.126 mg/Serving Size
**Thiamin by Fluorometric Method**	
Thiamin	0.030 mg/Serving Size
**Folic Acid by Microbiological Method**	
Folates (may contain folic acid)	2.83 mcg/Serving Size
**Ash**	
Ash	0.516 g/Serving Size
**Moisture by M100_T100**	
Moisture	1.21 g/Serving Size
**Total Polyphenols** *	
Total Polyphenols (Gallic Acid Equivalents)	105 mg/Serving Size
**ORAC** *	
ORAC	62.5 umol TE/g

Supplementary Table S4. Microbiological Analysis of Freeze-Dried Table Grape Powder

**Microorganism Analyzed**	**Result**	**Units**
Enterobacteriaceae	< 10	CFU/g
Yeast	20	CFU/ml
Mold	50	CFU/ml
Coliform	<10	CFU/g
E. coli	Negative/25g	+/-
Listeria	Negative/25g	+/-
Salmonella (BAX) PCR detection	Negative/25g	+/-
Aerobic Plate Count	420	CFU/g

C. Supplementary Figure S1

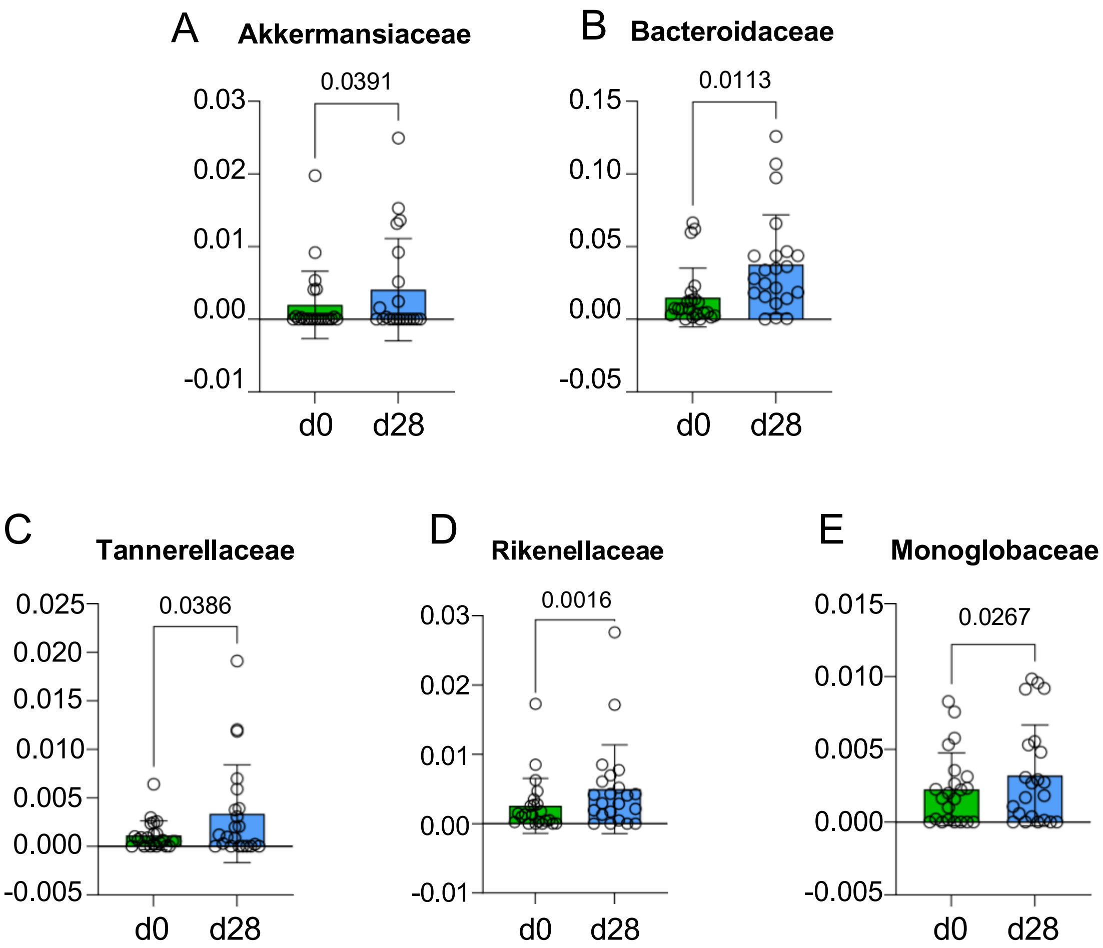

Supplementary Figure S1. Family-level analysis of taxa significantly different from day 0 to day 21 across the CD participants. P-value represents Wilcoxon matched-pairs signed rank test.
